# Strontium-Doped Hydroxyapatite as a Multifunctional Platform: Tailoring Structural, Mechanical, and Drug Delivery Properties for Bone-Related Applications

**DOI:** 10.1021/acsomega.6c05426

**Published:** 2026-09-21

**Authors:** Kairone de Paula Amaral, Luísa Arantes Fernandes Vieira, Jéssica Pauline Nunes Marinho, João Batista Santos Barbosa, André Felipe de Oliveira, Edésia Martins Barros de Sousa

**Affiliations:** † Comissão Nacional de Energia Nuclear − Centro de Desenvolvimento da Tecnologia Nuclear − (CNEN/CDTN), Laboratório de Materiais Nanoestruturados para Bioaplicações, Av. Presidente Antônio Carlos 6.627, Pampulha, 31270-901 Belo Horizonte, Minas Gerais, Brazil; ‡ Comissão Nacional de Energia Nuclear − Centro de Desenvolvimento da Tecnologia Nuclear − (CNEN/CDTN), SEMAV, Av. Presidente Antônio Carlos 6.627, Pampulha, 31270-901 Belo Horizonte, Minas Gerais, Brazil

## Abstract

Hydroxyapatite (HAp) is a calcium phosphate ceramic that has been extensively studied for its similarity to the mineral phase of bone tissue, and it exhibits excellent biocompatibility and osteoconductivity. However, its intrinsic brittleness and low fracture resistance limit its use in load-bearing environments, motivating the use of ionic doping to enhance its properties. Doping with strontium (Sr) is particularly advantageous, as this ion is associated with bone regeneration processes, improves osteogenic response, and favorably modifies the material’s lattice parameters and structural organization. In this work, Sr-doped HAp nanorods (5, 10, and 20%) were synthesized by the hydrothermal method, and their structural, mechanical, and drug delivery properties were investigated, with mechanical testing performed on non-sintered pellets to preserve the characteristics of the as-synthesized material. Drug incorporation and release assays with ciprofloxacin were conducted to evaluate the multifunctional potential for bone healing applications. The results confirmed the successful incorporation of Sr^2+^ into the hydroxyapatite lattice without the formation of secondary phases. Sr incorporation promoted lattice expansion, resulting in a maximum unit cell volume increase of 10 Å^3^, reduced nanorod length, and increased diffraction peak broadening, while Rietveld refinement revealed Sr occupancies of up to 19.5%. Furthermore, Sr incorporation increased the fracture toughness of the compacted non-sintered materials by up to 385% compared with pure HAp, while preserving the hydroxyapatite structure and enabling efficient ciprofloxacin loading and sustained release. Overall, Sr-doped HAp nanorods demonstrate tunable mechanical and functional properties, highlighting their potential as multifunctional materials for bone-related applications requiring both structural performance and localized therapeutic action.

## Graphical abstract

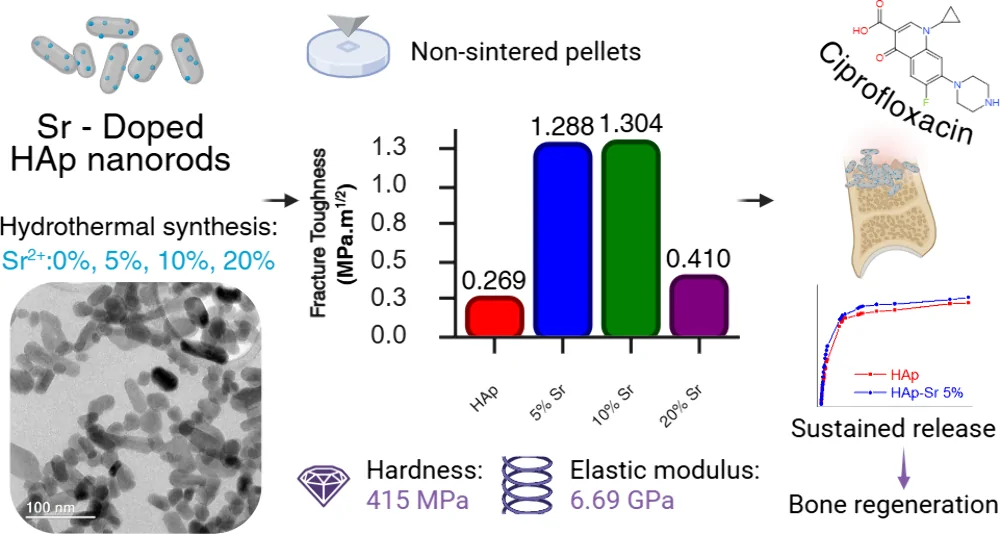

## 1 Introduction

The development of biomaterials capable of simultaneously providing mechanical support and biological functionality remains a major challenge in bone tissue engineering.^1,2^ Hydroxyapatite (HAp), a calcium phosphate ceramic that closely resembles the mineral phase of bone, has been extensively investigated due to its excellent biocompatibility, osteoconductivity, and chemical stability.^3,4^ However, its intrinsic brittleness and limited fracture resistance restrict its application in load-bearing environments, requiring strategies to enhance its structural properties without compromising bioactivity.^3,5^

One widely explored approach to overcoming these limitations is ionic substitution within the HAp lattice. The apatite structure allows substitution of Ca^2+^ by several divalent cations, including Sr^2+^, without altering its hexagonal crystal structure.^6–9^ Among these, strontium has attracted particular attention due to its biological relevance, being associated with bone regeneration processes and improved osteogenic response.^10–13^ In addition to its biological effects, Sr incorporation modifies lattice parameters and structural organization, influencing crystallinity and defect formation in hydroxyapatite.^9^

Beyond structural performance, the incorporation of therapeutic functionalities into biomaterials has become increasingly relevant. Hydroxyapatite has been widely explored as a drug delivery platform, particularly for antibiotics such as ciprofloxacin, which is commonly used in the treatment of bone-related infections. Systems based on HAp have demonstrated the ability to act as carriers for controlled drug delivery, combining structural support with therapeutic functionality.^14,15^

In addition to compositional modifications, the processing route plays a critical role in determining the final properties of ceramic biomaterials. Conventional densification methods, such as sintering, are often employed to improve mechanical performance; however, these processes can significantly alter the microstructure, reduce porosity, and affect surface chemistry.^16,17^ In contrast, the evaluation of materials in their green compacted state (non-sintered pellets) enables the investigation of intrinsic material behavior, preserving features such as higher surface area and structural defects that are particularly relevant for ion exchange, bioactivity, and drug incorporation.^16,18^ Furthermore, avoiding high-temperature processing represents an energetically favorable approach, contributing to more sustainable and cost-effective material development strategies.

From a biomedical standpoint, the use of non-sintered systems may also be advantageous in applications where partial resorption, higher reactivity, and controlled ion release are desirable, such as bone fillers, coatings, or temporary scaffolds.^4^ In this context, understanding the mechanical and functional behavior of compacted, non-sintered HAp-based systems provides valuable insight into their potential performance before densification or even as standalone materials in specific applications.

Nevertheless, a comprehensive understanding of how dopant concentration simultaneously influences structural features, mechanical behavior, and drug delivery performance in non-sintered HAp-based systems remains lacking. In particular, the identification of an optimal doping level that balances mechanical enhancement, structural stability, and controlled ion and drug release remains a key challenge for the development of multifunctional biomaterials. Some studies have employed a broad range of Sr substitution ratios, typically between 3 and 20 mol %, to tailor the physicochemical and biological properties of hydroxyapatite.^19–22^ Lower substitution levels (approximately 3–10 mol %) have been associated with enhanced osteoblast activity while preserving the apatite crystal structure, whereas higher substitutions (up to 20 mol %) have been investigated to maximize therapeutic Sr^2+^ release and further improve osteogenic performance. More recently, Li et al. systematically evaluated Sr substitutions from 1 to 100 mol % and demonstrated that approximately 20 mol % provided the best balance between structural stability, biocompatibility, osteogenic differentiation, angiogenesis, and immunomodulatory effects. Conversely, excessive Sr incorporation may alter the crystal structure, modify dissolution behavior, and potentially compromise biological performance. Therefore, evaluating intermediate substitution levels is essential for understanding the concentration-dependent effects of Sr incorporation and identifying compositions that balance structural integrity with functional performance.^23,24^

In this work, HAp nanorods were synthesized via a hydrothermal method and doped with Sr at different concentrations (5%, 10%, and 20%). These compositions were intentionally selected to investigate the concentration-dependent effects on the structural and mechanical properties of hydroxyapatite. Additionally, ciprofloxacin was incorporated to evaluate the drug loading capacity and release behavior. The study focuses on the evaluation of compacted, non-sintered pellets, aiming to provide insight into the intrinsic behavior of the material under conditions that preserve its physicochemical reactivity while evaluating its applicability in the green state. Rather than mimicking the elemental composition of natural bone, this study explores strontium as a functional dopant to tailor the properties of hydroxyapatite, aiming to develop a multifunctional platform that combines tunable structural and mechanical characteristics with drug delivery capability for bone-related applications.

## 2 Experimental Section

### 2.1 Materials

All chemicals were acquired from Sigma-Aldrich. Hydroxyapatite was synthesized using calcium nitrate tetrahydrate (Ca­(NO_3_)_2_·4H_2_O), strontium nitrate (Sr­(NO_3_)_2_), potassium phosphate (K_2_HPO_4_·3H_2_O), hexadecyltrimethylammonium bromide (CTAB), and ammonium hydroxide (NH_4_OH).

### 2.2 Hydroxyapatite Synthesis

Hydroxyapatite (HAp) nanorods were synthesized via the hydrothermal method, as previously reported,^25,26^ using aqueous solutions containing calcium and phosphate precursors in stoichiometric proportions. Strontium doping was carried out by partially substituting Ca^2+^ with Sr^2+^ at molar ratios of 5, 10, and 20%.

The precursor solutions containing calcium nitrate tetrahydrate and strontium nitrate were added dropwise under continuous stirring with dibasic potassium phosphate, and the pH was adjusted using NH_4_OH. After 14 h of aging, the resulting mixture was transferred to a hydrothermal reactor and subjected to a controlled temperature for 10 h, followed by washing and drying steps

#### 2.2.1 Ciprofloxacin Incorporation

Drug incorporation was carried out by initially preparing a ciprofloxacin solution at a concentration of 1 mg mL^–1^. Due to its low solubility at neutral pH, the drug was dissolved by adjusting the pH to 2 using a 2 mol L^–1^ HCl solution. After complete dissolution, the pH was adjusted to 4 using a 1 mol L^–1^ NaOH solution.

The materials (HAp and HAp-Sr 5%) were added to the ciprofloxacin solution at a 1:1 mass ratio. After incorporation, the samples were filtered and dried at room temperature. The amount of incorporated drug was determined by thermogravimetric analysis (TGA) and CHN elemental analysis.

### 2.3 Characterization

Phase composition and crystallinity were analyzed by X-ray diffraction (XRD) with Cu Kα (λ = 0.154 nm) at 0.8 kW from 4° to 120° (2θ) at a scanning rate of ∼1°/min (Ultima IV, Rigaku, Japan). Powder samples were gently compressed onto the sample holder to obtain a flat surface for XRD analysis.

Fourier transform infrared (FTIR) spectra were obtained using a VERTEX 70v spectrometer (Bruker, Germany), operating under vacuum and equipped with an Nd:YAG laser source (λ = 1064 nm). The measurements were carried out in the range of 4000–400 cm^–1^, with a spectral resolution of 4 cm^–1^ and 128 scans. The analyses were performed in attenuated total reflectance (ATR) mode using a diamond crystal. Complementary infrared analyses were performed by diffuse reflectance infrared Fourier transform spectroscopy (DRIFTS) using a Harrick accessory coupled to a Bruker FTIR-70 V spectrometer. The measurements were conducted in the spectral range of 4000–400 cm^–1^. Before analysis, the samples were manually ground in an agate mortar and sieved through a 38 μm mesh to ensure uniform particle size. The powders were then mixed with KBr to minimize concentration effects and improve spectral quality.

Fourier transform Raman (FT-Raman) spectra were acquired using a RAM-II module coupled to an FTIR spectrometer. Measurements were performed in the range of 3500–50 cm^–1^ with a spectral resolution of 4 cm^–1^ and a laser power of 100 mW.

The morphology and average size distribution of the synthesized materials were analyzed by scanning electron microscopy (SEM) using a Sigma series microscope (ZEISS, Germany); for elemental mapping, a FEI Quanta 200 FEG (JEOL, Japan) was used, and transmission electron microscopy (TEM) images were acquired using a FEI Tecnai G2-20 SuperTwin (Thermo Fisher Scientific, USA). For sample preparation, the materials were dispersed in Milli-Q water at a concentration of 0.1 mg mL^–1^ and sonicated using a probe ultrasonicator with an energy input of 15 kJ. A drop of the resulting dispersion was deposited onto a silicon wafer and allowed to dry. Before analysis, the samples were coated with a thin gold layer to improve conductivity. The elemental composition of doped samples was evaluated by energy-dispersive X-ray spectroscopy (EDS) using an XFlash 410-M detector (Bruker, USA), coupled to the SEM system. Particle size measurements were performed using ImageJ software after calibration with the scale bar provided in each SEM micrograph. The particle length was defined as the maximum dimension of each particle. Measurements were obtained from different image fields to ensure statistical representativeness, with more than 50 particles analyzed for each sample. The average particle lengths reported correspond to the arithmetic mean of these individual measurements. Statistical analysis was subsequently performed using Minitab software.

The carbon, hydrogen, and nitrogen contents of the samples were quantified by elemental analysis using an EA 2400 Series II CHNS/O analyzer (PerkinElmer, USA). Approximately 2–3 mg of each sample was used for the measurements.

#### 2.3.1 Stability Assay

The stability of the doped materials was evaluated by preparing aqueous suspensions at different time intervals. HAp-Sr samples (5, 10, and 20%) were dispersed in simulated body fluid (SBF) at a concentration of 1 mg mL^–1^. The suspensions were incubated under constant agitation (50 rpm) at 37 °C for 24 and 48 h. After incubation, the samples were filtered using a Swinnex system equipped with a nylon membrane (450 nm pore size). The filtrates were analyzed by inductively coupled plasma optical emission spectrometry (ICP-OES) using an ARCOS FHE12 instrument (SPECTRO Analytical Instruments, Germany) to quantify the amount of Sr released from the HAp matrix.

#### 2.3.2 Mechanical Properties

Vickers microhardness tests were performed to determine the hardness of the samples, following the ASTM C1327-15 standard. Pellets with a diameter of 11 mm and a thickness of approximately 3 mm were prepared by uniaxial pressing of 700 mg of powder using a hydraulic press (Ciola), applying a load of 6 ton cm^–2^ for 6 s. The pellets were produced in duplicate.

Microhardness measurements were carried out using a Universal Hardness Tester (930N, Wolpert Wilson Instruments, USA) under a load of 0.5 kgf with a dwell time of 10 s. Each pellet was indented at three different locations on the flat surface of the compacted specimen. The indentations were performed at least 2 mm away from the pellet edge, and were distributed over the central region with the maximum possible spacing between them to minimize boundary effects and the influence of neighboring indentations. Scanning electron microscopy (SEM) was used to accurately visualize and measure the indentation marks.

The Vickers hardness (*HV*) was calculated according to

1 $$HV=1.8544\left(\frac{P}{d^{2}}\right)$$

where *HV* is the Vickers microhardness (kgf mm^–2^), *P* is the applied load (kgf), and *d* is the average diagonal length of the indentation (mm).

Fracture toughness (*K_IC_*) was estimated from the same indentations using SEM images, based on the radial crack lengths, according to^27^

2 $$K_{IC}=0.203HVa^{2}c^{-3/2}$$

Here *HV* is the Vickers hardness (GPa), *K_IC_* is the fracture toughness (MPa·m^1^/^2^), *a* is half of the indentation diagonal (μm), and *c* is half of the radial crack length (μm).

Nanoindentation tests were performed using a scanning probe microscope coupled with a nanoindenter (MFP-3D-AS, Asylum Research) to determine the elastic modulus of the samples. The measurements were carried out on pellets prepared using the same procedure described for the Vickers microhardness tests. The elastic modulus was calculated by the instrument software using the Oliver–Pharr method. For each sample, 16 indentations were performed in a regular 4 by 4 grid with sufficient spacing between adjacent indentations to avoid interactions between the deformation zones, ensuring statistically reliable measurements.

#### 2.3.3 Ciprofloxacin *In Vitro* Release

*In vitro* release studies were performed using a dialysis method. Approximately 2 mg of drug-loaded material was placed into a dialysis membrane (D9277, Sigma-Aldrich, USA) and immersed in 40 mL of simulated body fluid (SBF) in a previously sterilized polyethylene flask. The system was maintained at 37 °C under constant agitation (50 rpm). Drug release over time was monitored by UV–Vis spectroscopy (Shimadzu UV-2400), measuring the absorbance at 272 nm, and the concentration was determined using a calibration curve.

The fraction of the drug released was calculated according to

3 $$\frac{m}{m_{0}}=\frac{C\cdot V}{m_{0}}$$

where *m* is the amount of drug released at time *t*, *m*_*0*_ is the initial amount of drug incorporated, *C* is the measured concentration, and *V* is the volume of the release medium (40 mL).

The release kinetics were evaluated using conventional mathematical models, as described below:

Zero-order model:

4 $$\frac{m}{m_{0}}=K_{0}t$$

First-order model:

5 $$\frac{m}{m_{0}}=1-e_{1}^{-K_{1}t}$$

Higuchi model:

6 $$\frac{m}{m_{0}}=K_{H}t^{1/2}$$

Korsmeyer–Peppas model:

7 $$\frac{m}{m_{0}}=Kt^{n}$$

where *K*_*0*_, *K*_*1*_, *K*_*H*_, and *K* are the release constants for the zero-order, first-order, Higuchi, and Korsmeyer–Peppas models, respectively, *n* is the release exponent, and *m/m*_*0*_ is the fraction of drug released at time *t*.

## 3 Results and Discussion

The X-ray diffraction patterns of HAp and Sr-doped samples (HAp-Sr 5%, 10%, and 20%) are presented in Figure 1. All samples exhibited diffraction peaks characteristic of hydroxyapatite, consistent with the hexagonal crystal structure (space group *P6*_*3*_ */m*), indicating the successful synthesis of the desired phase.^28^

**Figure 1:**
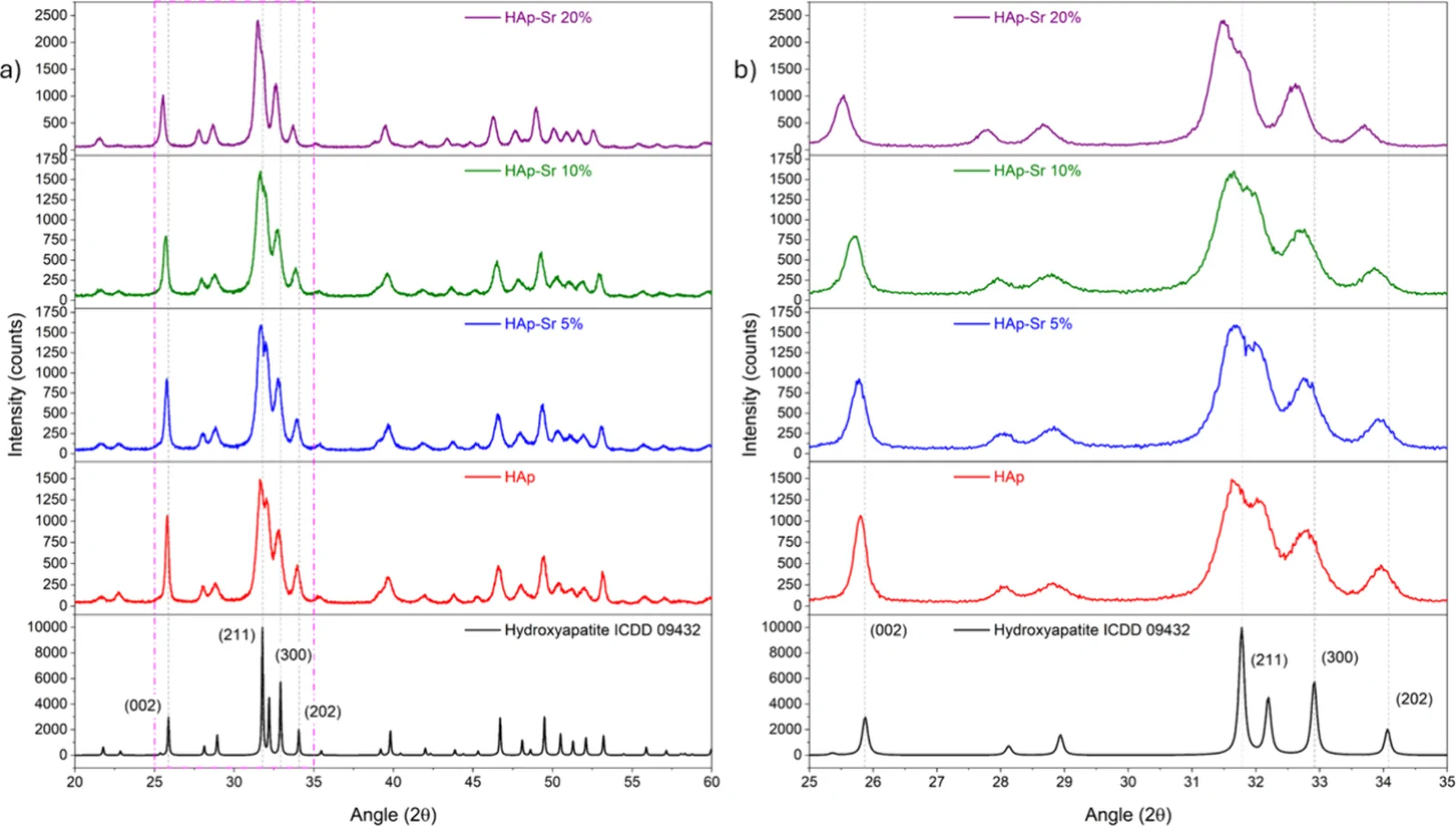
a) XRD diffraction patterns of HAp, HAp-Sr5%, HAp-Sr10%, and HAp-Sr20%: (a) full diffraction patterns and (b) enlarged view of the 2θ region highlighted by the magenta dashed box in (a).

The main reflections observed at 2θ approximately 25.9°, 31.8°, 32.9°, and 34.0° are attributed to the (002), (211), (300), and (202) planes of HAp, respectively.^28^ A progressive shift of the diffraction peaks toward lower 2θ values was observed with increasing Sr content. Specifically, the (002) reflection, presented in Table 1, shifted from 25.80° for pure HAp to 25.77°, 25.70°, and 25.53° for HAp-Sr 5%, HAp-Sr 10%, and HAp-Sr 20%, respectively. trend is consistent with the findings of Frasnelli et al. and Zhou et al.^29,30^ Frasnelli et al. reported a progressive shift of approximately 0.20° between pure HAp and HAp doped with 10% Sr, with even greater displacements observed as the Sr content increased up to 100%. This behavior is attributed to the substitution of Ca^2+^ ions (ionic radius ∼0.99 Å) by the larger Sr^2+^ ions (∼1.12 Å), resulting in the expansion of the HAp lattice parameters, as widely reported in the literature.^7,28,29,31^ Furthermore, the full width at half maximum (FWHM) values for the (002) reflection presented in Table 1 show a progressive peak broadening with increasing Sr content, evidenced by the increase in FWHM from 0.206 for pure HAp to 0.264, 0.290, and 0.267 for HAp-Sr 5%, HAp-Sr 10%, and HAp-Sr 20%, respectively. This behavior indicates that Sr incorporation induces structural disorder and lattice distortions within the hydroxyapatite crystal structure.^29,30,32^ Moreover, no secondary phases related to calcium oxide, tricalcium phosphate, or strontium-containing compounds were detected within the resolution of the technique, indicating that Sr^2+^ ions were incorporated into the hydroxyapatite lattice without the formation of detectable secondary crystalline phases. This incorporation is accompanied by changes in the lattice parameters, diffraction peak positions, and peak broadening, as confirmed by the Rietveld refinement and FWHM analysis. Rietveld refinement (Figure 2 and Table 2) further demonstrated that Sr^2+^ occupies substitutional Ca^2+^ sites rather than interstitial positions, as evidenced by the refined site occupancies, which closely match the nominal Sr contents, while preserving the hydroxyapatite crystal structure.

**Table 1:** Position of the (002) Diffraction Peak (2θ) and Its Full Width at Half Maximum (FWHM) for HAp and Sr-Doped HAp Samples

**Sample**	**2θ at (002) peak (deg)**	**FWHM**
**HAp**	25.80	0.206
**HAp-Sr 5%**	25.77	0.264
**HAp-Sr 10%**	25.70	0.290
**HAp-Sr 20%**	25.53	0.267

**Figure 2:**
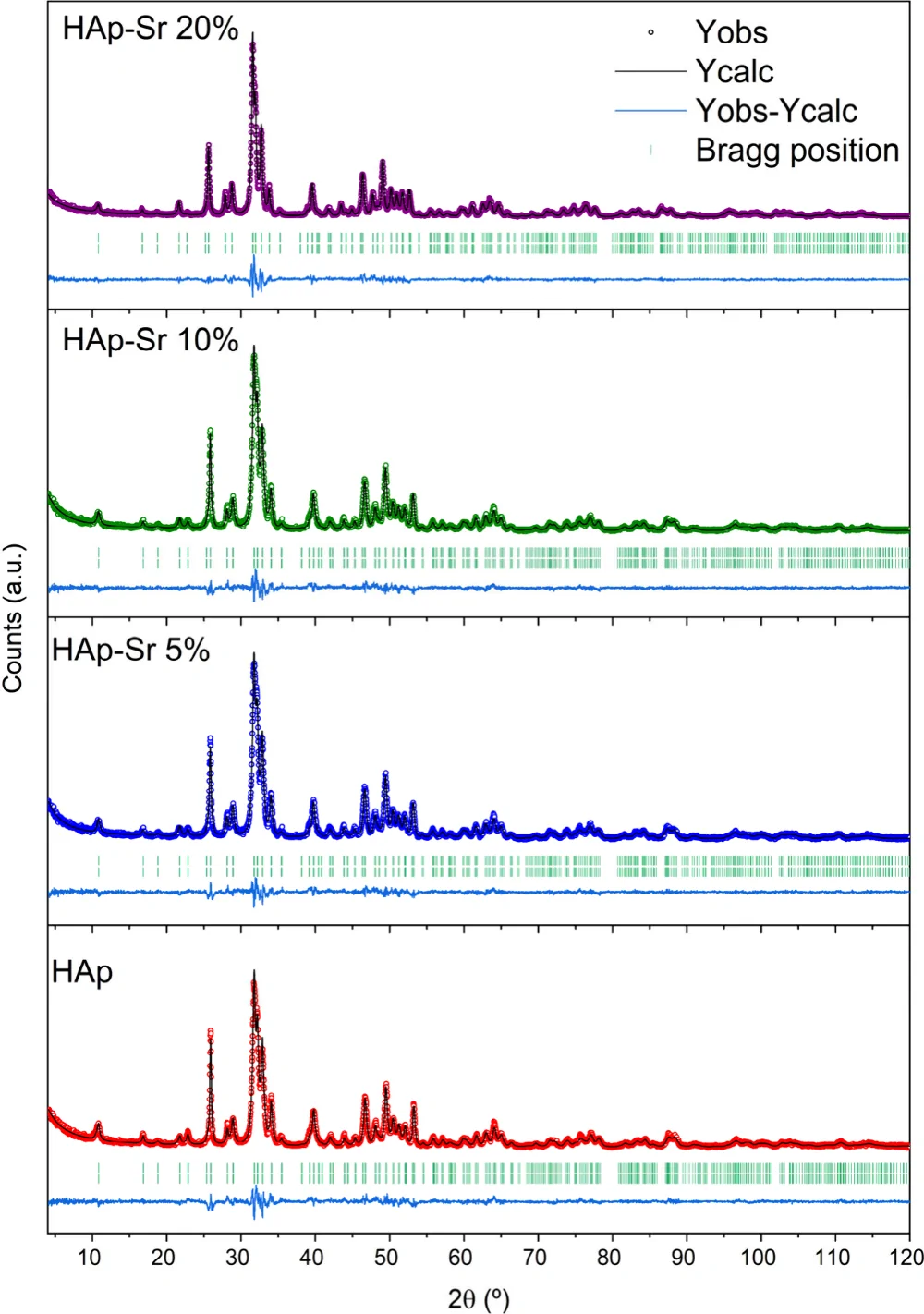
XRD patterns of the HAp, HAp-Sr 5%, HAp-Sr 10%, and HAp-Sr 20% along with the Rietveld refinement results. The measured intensities (*Y*_obs_) are represented by circles, while the calculated pattern (*Y*_calc_) is represented by the black curves, and the difference between the calculated and observed intensities (*Y*_calc_ – *Y*_obs_) is represented by the blue curves. The expected Bragg positions of each phase are represented by vertical markers.

**Table 2:** Unit Cell Parameters Obtained via Rietveld Refinement of Pure and Sr-Doped Samples^a^

	**Unit cell parameters**				**Reliability Factors**		
Sample	*a = b* (Å)	*c* (Å)	*V* (Å^3^)	Sr occupancy (%)	χ^2^	R_p_	R_wp_
**HAp**	9.43283(46)	6.87856(36)	530.044(46)	—	1.39	9.20	12.0
**HAp-Sr 5%**	9.43894(28)	6.89142(23)	531.724(29)	4.6	1.27	8.5	11.2
**HAp-Sr 10%**	9.44851(49)	6.90392(40)	533.768(50)	10.3	1.30	13.8	16.2
**HAp-Sr 20%**	9.47887(39)	6.95195(31)	540.942(40)	19.5	1.47	13.7	15.7

a Sr occupancy obtained in relation to calcium (Sr/Ca).

Figure 2 shows the XRD patterns and Rietveld refinement results for all samples at room temperature. During refinement, it was observed that the profile functions could not adequately fit the peak corresponding to the (002) plane, suggesting the presence of preferred orientation. This result is consistent with literature reports indicating that hydroxyapatite nanoparticles synthesized via the hydrothermal method exhibit preferential growth along the crystallographic c-axis, which favors the preferred orientation associated with the (002) plane.^33,34^ A technique was therefore employed involving two identical phases with identical values for all parameters except the profile parameters. These were left free so that, in combination with the preferred orientation parameter, they would result in a superior overall fit. Comparing the doped samples with pure hydroxyapatite reveals that increasing the Sr content leads to a potential reduction in crystallinity or the formation of smaller nanoparticles, resulting in progressive peak broadening—as evidenced by the FWHM values presented in Table 1.^35^

In nanocrystalline hydroxyapatite, the width of the diffraction peaks is strongly influenced by crystallite size and lattice disorder, and broad reflections do not necessarily indicate poor crystallinity. Londoño-Restrepo et al. further demonstrated by HRTEM that highly ordered hydroxyapatite nanocrystals may still exhibit broad XRD peaks due to the simultaneous contributions of elastic and inelastic scattering.^36^

In other studies like Frasnelli and collaborators (2017), the shape of the (002) peak reduced, and the relative intensity of the (002) peak decreased, as well as the c-axis texture being more equiaxed or thinner.^29^ In this study, the reduction of the (002) peak intensity with Sr-doped HAp materials was attributed to the presence of smaller, more strained, and less crystalline nanoparticles, as well as an altered preferred orientation.^35,37^ As shown in Table 2, an increase in strontium content in hydroxyapatite leads to an increase in cell parameters, including the unit cell volume. The alteration of these parameters is related to distortion in the crystal lattice caused by the partial substitution of Ca^2+^ ions with Sr^2+^ ions, which have a larger ionic radius.^29,30,35^ Several studies have confirmed this trend through Rietveld refinement.^28–30,35,37^ An increase in the atomic occupation of strontium in the hydroxyapatite lattice was also observed, approaching the nominal amount added during synthesis of the nanocomposites by 5%, 10%, and 20%, obtaining 4.6%, 10.3%, and 19.5%, respectively. Therefore, XRD and Rietveld refinement revealed that doping with large amounts of strontium altered the lattice parameters, the shape of the characteristic peaks, and the degree of crystallinity of the nanocomposites.

The IR spectra of HAp and Sr-doped samples (HAp-Sr 5%, 10%, and 20%) are shown in Figure 3a and summarized in Table 3. All samples exhibited the characteristic vibrational bands of hydroxyapatite, associated with phosphate groups. The bands at approximately 1089 and 1026 cm^–1^ are assigned to the asymmetric stretching vibrations of PO_4_^3–^, while the band at 962 cm^–1^ is attributed to the symmetric stretching vibration. The bands at 601 and 563 cm^–1^ are assigned to the bending vibrations of the phosphate group, whereas the band at 472 cm^–1^ is attributed to the asymmetric bending mode. Additionally, the band at approximately 630 cm^–1^ is assigned to the stretching vibration of structural hydroxyl (OH^‑^) groups.^38–40^ Carbonate-related bands at approximately 1454 and 1415 cm^–1^, associated with carbonate vibrations, together with the band at approximately 875 cm^–1^, attributed to the out-of-plane bending vibration of CO_3_^2–^, were more evident in undoped HAp, indicating the formation of B-type carbonated hydroxyapatite.^41–44^ The reduced intensity or absence of these bands in Sr-doped samples suggests that Sr incorporation decreases the presence of CO_3_^2–^ ions in the HAp structure, likely due to lattice distortions that hinder carbonate substitution.^45^

**Table 3:** Assignment of the IR and Raman Bands Observed for HAp and Sr-Doped HAp Samples, Including the Corresponding Vibrational Modes and Literature References

**Wavenumber (cm** ^**‑1**^ **)**	**Technique**	**Vibrational mode**	**Functional group/Assignment**
962	IR/Raman	symmetric stretching	PO_4_^3–^
472	IR	bending	PO_4_^3–^
1089	IR	asymmetric stretching	PO_4_^3–^
1026			
1074	Raman	asymmetric stretching	PO_4_^3–^
1047			
601	IR	bending	PO_4_^3–^
563			
630	IR	OH^–^ stretching	Structural hydroxyl
875	IR	out-of-plane bending	CO_3_^2^
1454	IR	asymmetric stretching	CO_3_^2–^
1415			

**Figure 3:**
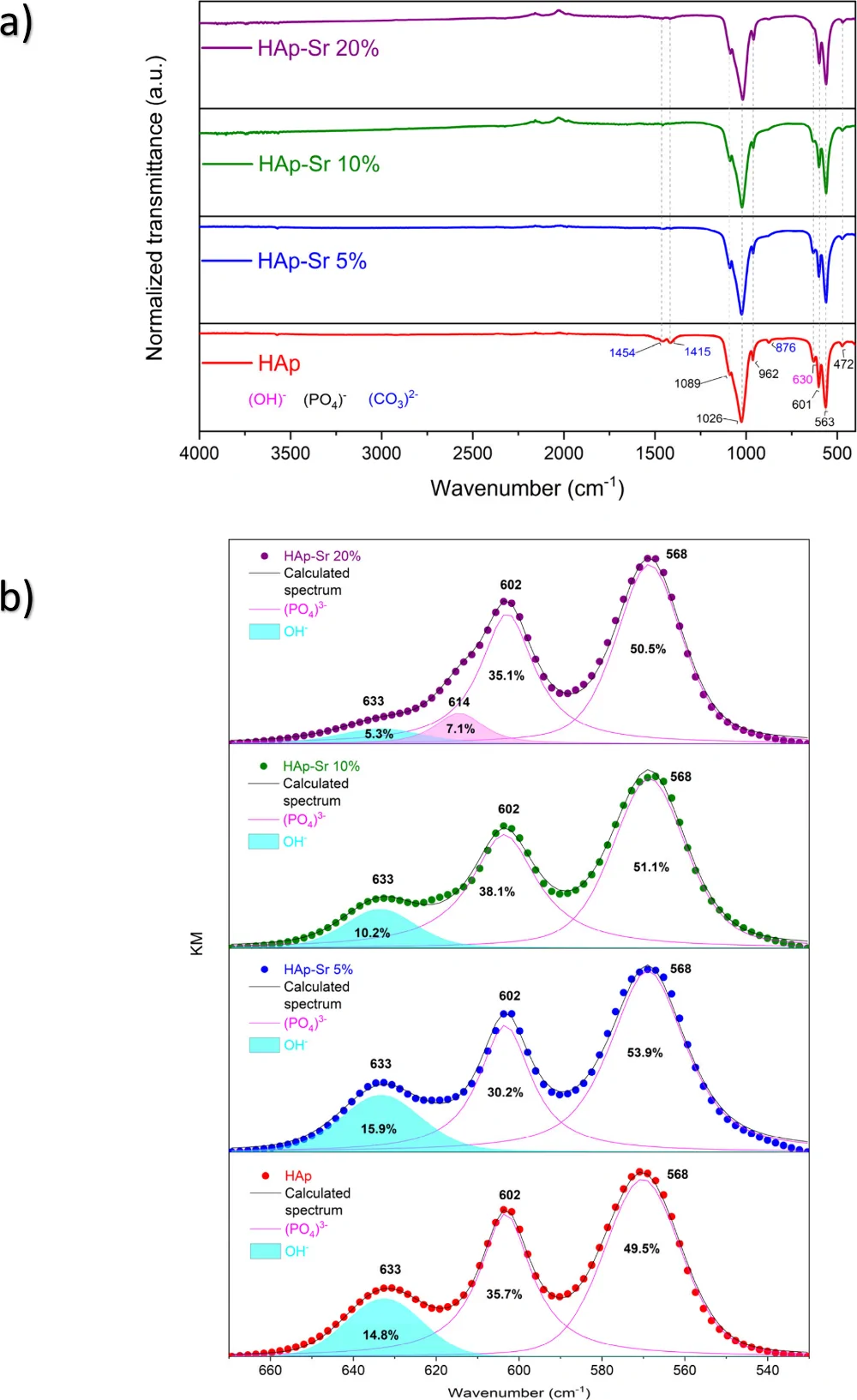
a) FTIR - ATR spectra of HAp, HAp-Sr 5%, HAp-Sr 10%, and HAp-Sr 20% and b) DRIFTS spectra deconvolution of the samples in the region of 540 to 670 cm^–1^.

Diffuse reflectance Fourier-transform infrared spectroscopy (DRIFTS) was employed in this study due to its greater suitability for characterizing particulate solid materials, such as hydroxyapatite nanoparticles. Compared to conventional transmission FTIR, which generally requires the preparation of KBr pellets and can introduce variabilities associated with sample homogenization, moisture absorption, and pressure-induced structural changes during compaction, the DRIFTS technique has the advantage of requiring minimal sample preparation, thereby better preserving the sample’s original physicochemical characteristics. Furthermore, unlike FTIR-ATR, whose analysis is predominantly limited to the most superficial layers of the material, DRIFTS allows for a more representative investigation of the total sample volume, making it particularly advantageous for heterogeneous systems and powdered materials. For quantitative analysis, the reflectance spectra were converted to Kubelka-Munk (KM) units, since this function exhibits a linear relationship with the concentration of the functional groups present. This conversion provides a more robust and reliable correlation between the intensity of the vibrational bands and the chemical composition of the surface, facilitating the comparison between different formulations and the evaluation of structural changes resulting from strontium doping.^46^

The DRIFTS spectra obtained for the samples of pure hydroxyapatite (HAp) and strontium-substituted hydroxyapatite (HAp-Sr 5%, HAp-Sr 10%, and HAp-Sr 20%) in the 540 – 670 cm^–1^ region, shown in Figure 3b, showed that the characteristic vibrational bands of the apatitic phase were maintained in all formulations, indicating that the incorporation of Sr^2+^ does not compromise the formation of the hydroxyapatite crystal structure, in agreement with studies of Sr-HAp in which the apatitic phase is preserved even with increased Sr content.^7^

In all samples, bands centered at approximately 568 cm^–1^ and 602–614 cm^–1^ were observed, attributed to the deformation modes of the phosphate group (PO_4_^3–^), in addition to the band around 633 cm^–1^, related to the vibrational mode of the hydroxyl groups (OH^‑^) in the structural channels of HAp, in good agreement with the classical assignments for apatites.^40^

The pure HAp sample exhibited relative areas of 49.5% (568 cm^–1^), 35.7% (602 cm^–1^), and 14.8% (633 cm^–1^). With the incorporation of Sr, gradual changes in the distribution of the DRIFTS band areas were observed, indicating modifications in the local vibrational environment of the phosphate and hydroxyl groups. These observations are consistent with the lattice expansion and structural changes revealed by the Rietveld refinement of the XRD data, as well as with the increased peak broadening determined from the FWHM analysis.

In the HAp-Sr 5% sample, there was a slight increase in the band area at 568 cm^–1^ (53.9%) and a slight increase in the contribution of the OH^‑^ group to 15.9%, suggesting minor structural disturbances. For HAp-Sr 10%, a reduction in the OH^‑^ band area to 10.2% was observed, accompanied by an increase in the band area at 602 cm^–1^ (38.1%), indicating greater local disorder in the crystal lattice.^7,47^

Terra et al. observed that an increase in Sr is associated with greater disorder and the loss or alteration of OH^‑^ groups, as detected by FTIR and EXAFS.^7^ O’Donnell *et al*. and Baldassarre *et al*. showed that the addition of Sr alters the local structure and crystallinity, in parallel with subtle changes in the phosphate bands in FTIR/Raman.^32,48^

The HAp-Sr 20% sample exhibited the most significant changes, with a marked reduction in the band at 633 cm^–1^ to 5.3% and the emergence of a new contribution at 614 cm^–1^ (7.1%), suggesting greater distortion in the vibrational environment of the phosphate group and a possible increase in structural heterogeneity. According to studies reported in the literature, the presence of a band at 614 cm^–1^ in apatites is linked to a labile phosphate environment and low crystallinity, frequently observed in biological or poorly matured apatites that have not reached a high degree of crystalline perfection.^49,50^

From a structural standpoint, these changes are consistent with the literature on strontium-doped hydroxyapatite, which reports that the isomorphous substitution of Ca^2+^ with Sr^2+^ causes lattice expansion, increased local disorder, and changes in the vibrational modes of the phosphate group. The shift and redistribution of the band areas are often associated with a modification of the local symmetry of the PO_4_^3–^ tetrahedra, while the progressive narrowing of the OH^‑^ band indicates a disruption of the structural channels along the *c*-axis.^7,29,51^

Altogether, the DRIFTS results demonstrate that Sr doping promotes gradual changes in the chemical environment of hydroxyapatite, maintaining the apatitic phase while inducing progressive changes in the structural organization of the phosphate and hydroxyl groups, becoming more pronounced at the highest doping level (20% Sr).

The FT-Raman spectra of HAp and Sr-doped samples (HAp-Sr 5%, 10%, and 20%) are presented in Figure 4. The spectra exhibit the characteristic vibrational modes of phosphate groups, consistent with the FTIR results. The intense band at ∼962 cm^–1^ is attributed to the symmetric stretching mode of PO_4_^3–^, while the bands at ∼1047 and 1074 cm^–1^ correspond to the asymmetric stretching modes.^52^

**Figure 4:**
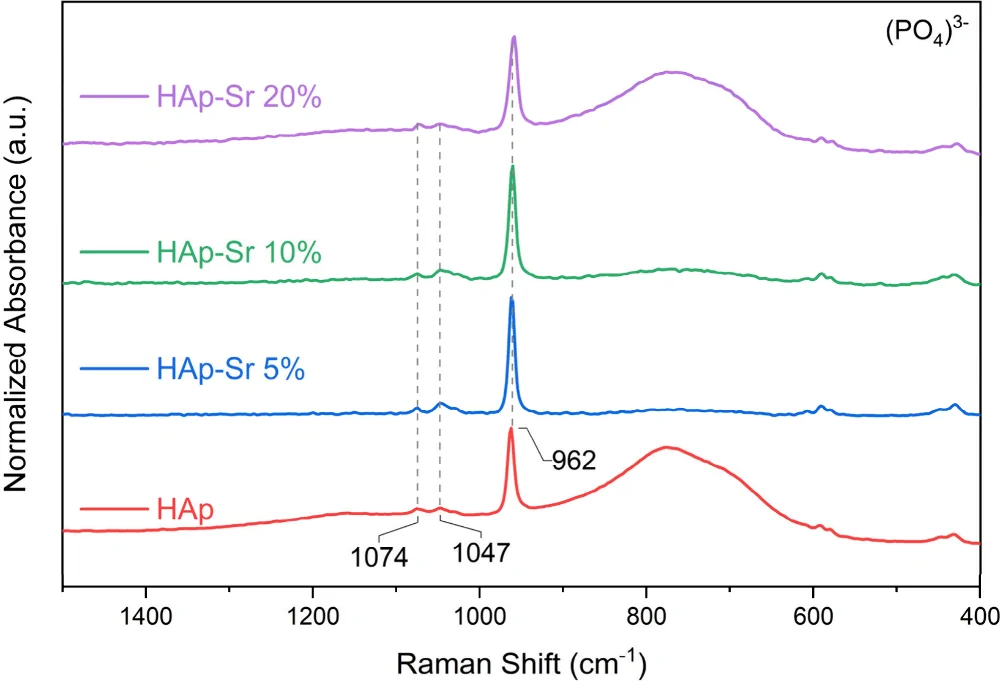
FT-Raman spectra of HAp, HAp-Sr 5%, HAp-Sr 10%, and HAp-Sr 20%.

The main PO_4_^3–^ band shifts from ∼962 cm^–1^ in HAp and HAp–Sr 5% to ∼960 cm^–1^ in HAp–Sr 10% and to ∼958 cm^–1^ in HAp–Sr 20%, evidencing a systematic shift toward lower wavenumbers with increasing Sr content. These shifts reflect changes in the local bonding environment and can be attributed to lattice expansion caused by the substitution of Ca^2+^ (0.99 Å) by larger Sr^2+^ ions (1.12 Å).^32,47,53^

Overall, the Raman results are consistent with FTIR data and confirm the presence of the characteristic phosphate bands in all samples, with subtle shifts observed at higher Sr contents, indicating increased structural disorder and lattice distortion. This behavior is further supported by the XRD results and Rietveld refinement, which reveal a systematic expansion of the unit cell with increasing Sr content.

SEM images of HAp and Sr-doped samples (HAp-Sr 5%, 10%, and 20%) are presented in Figure 5, along with their respective particle size distributions in Table 4.

**Figure 5:**
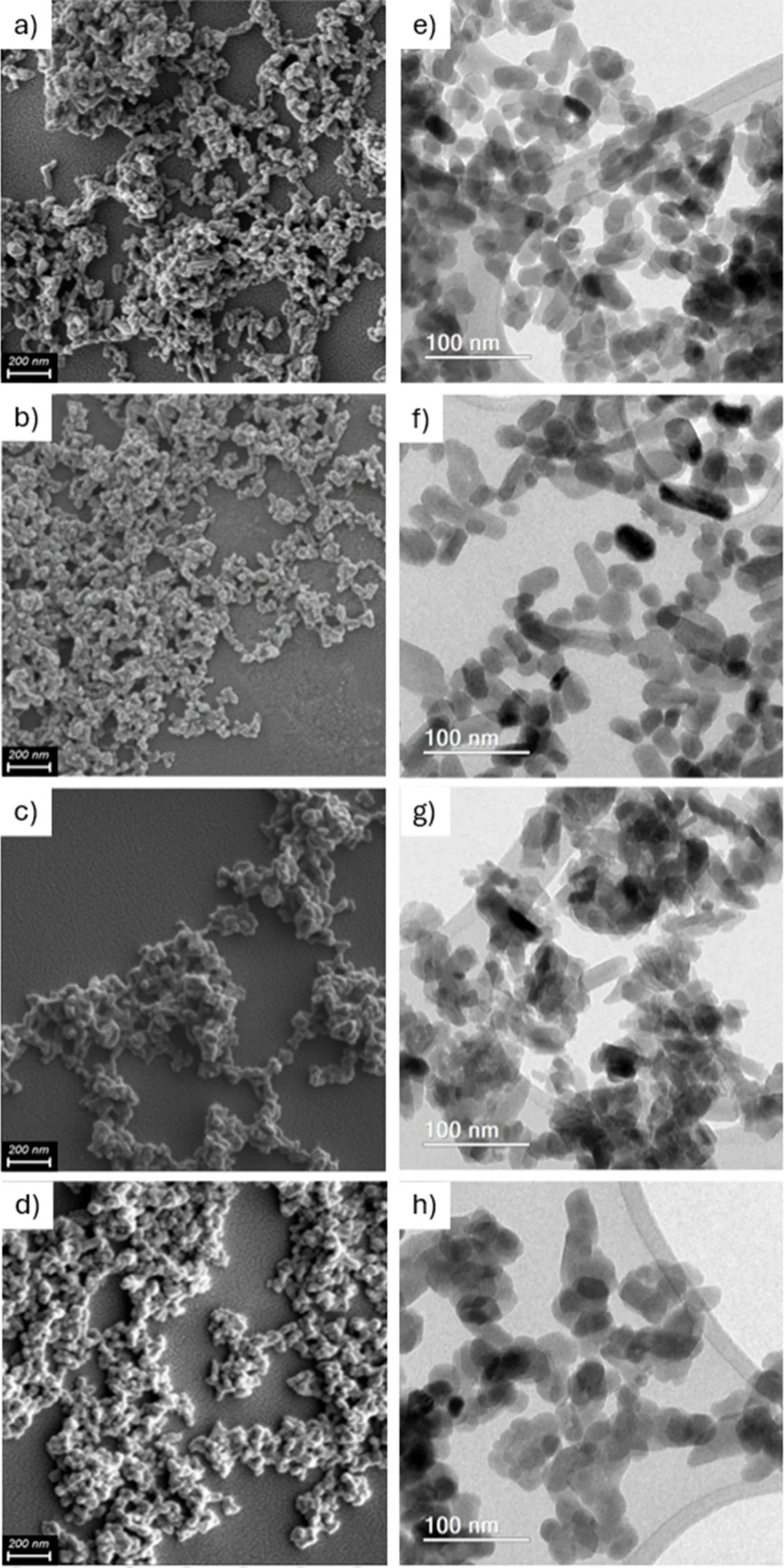
Representative SEM ((a–d), left column) and TEM ((e–h), right column) micrographs of the synthesized hydroxyapatite powders: (a,e) HAp, (b,f) HAp-Sr 5%, (c,g) HAp-Sr 10%, and (d,h) HAp-Sr 20%.

**Table 4:** Average Rod Length of HAp and Sr-Doped Hydroxyapatite Nanorods

**Sample**	**Average Rod Length (nm)**	**Number of measured particles**
HAp	97 ± 20	66
HAp-Sr 5%	84 ± 18	85
HAp-Sr 10%	55 ± 15	76
HAp-Sr 20%	63 ± 17	89

The SEM and TEM micrographs reveal that all samples exhibit a predominantly elongated morphology, characteristic of hydroxyapatite nanorods, with heterogeneous size and shape distributions and a tendency to form agglomerates. The observed agglomeration is commonly reported for hydroxyapatite nanoparticles and has been associated with their high surface energy.^31^

The formation and morphology of nanorods were strongly influenced by Sr doping. Baldassarre *et al*. associate the incorporation of larger Sr^2+^ ions into the HAp lattice with both the growth mechanism and the final dimensions of the particles.^32^ Additionally, increased dopant concentration resulted in more pronounced heterogeneity in both size and morphology.

The average particle lengths are summarized in Table 4. A clear trend of decreasing nanorod length with increasing Sr content was observed, with values of approximately 97 ± 20 nm for HAp, 84 ± 18 nm for HAp-Sr 5%, 55 ± 15 nm for HAp-Sr 10%, and 63 ± 17 nm for HAp-Sr 20%. This reduction is consistent with previous studies, which report that Sr incorporation into the HAp structure leads to morphological changes, resulting in shorter and more distorted crystals.^51,54^ Moreover, the reduction in nanorod dimensions is consistent with the broadening of the XRD peaks observed with increasing Sr content when compared to pure HAp. Taken together, the TEM and XRD results indicate that peak broadening is strongly influenced by the reduced dimensions of the nanostructures and the increased lattice disorder induced by Sr incorporation, rather than being solely indicative of reduced crystallinity.

Despite the size reduction, all samples exhibited particle dimensions within the range of 50 – 200 nm, which is considered favorable for biomedical applications. Materials within this size range present enhanced interaction with biological systems and improved cellular uptake compared to significantly smaller or larger particles.^55^

The EDS spectra of all samples are presented in Figure 6 and were used to evaluate the presence of strontium in the synthesized materials. The spectra exhibit the characteristic peaks of the elements that constitute hydroxyapatite, including Ca, P, and O, confirming the expected composition.

**Figure 6:**
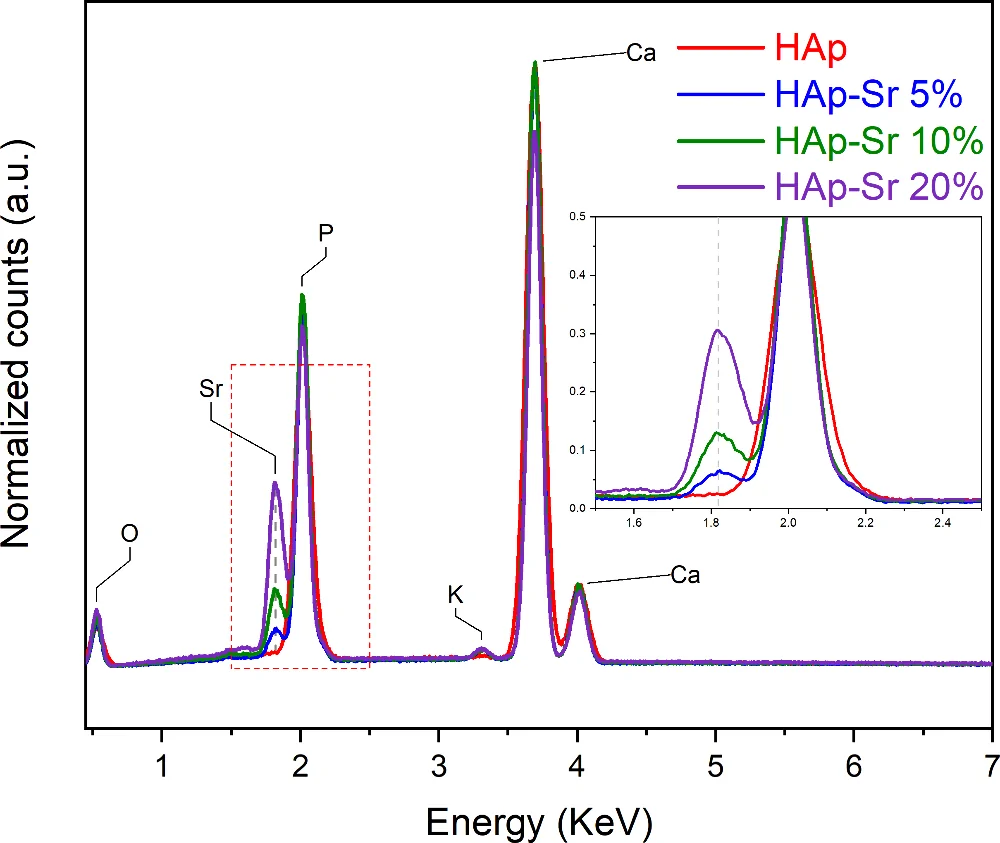
EDS spectra of HAp samples with an inset highlighting the Sr Lα peak region.

A magnified region highlights the characteristic Sr Lα emission at approximately 1.806 keV. The observation of this peak in all doped samples is consistent with the presence of Sr in the synthesized materials. Furthermore, an increase in peak intensity with increasing Sr content was observed, indicating that the relative amount of Sr in the samples is consistent with the nominal doping levels. A minor potassium (K) signal was also detected, likely associated with residual species from the synthesis process.

The EDS elemental maps, presented at Figure 7 further revealed a homogeneous distribution of Sr throughout the analyzed particles, with no evidence of localized Sr-rich regions or elemental segregation. This homogeneous distribution is consistent with the substitutional incorporation of Sr into the hydroxyapatite lattice inferred from the XRD, Rietveld refinement, and FWHM analyses.

**Figure 7:**
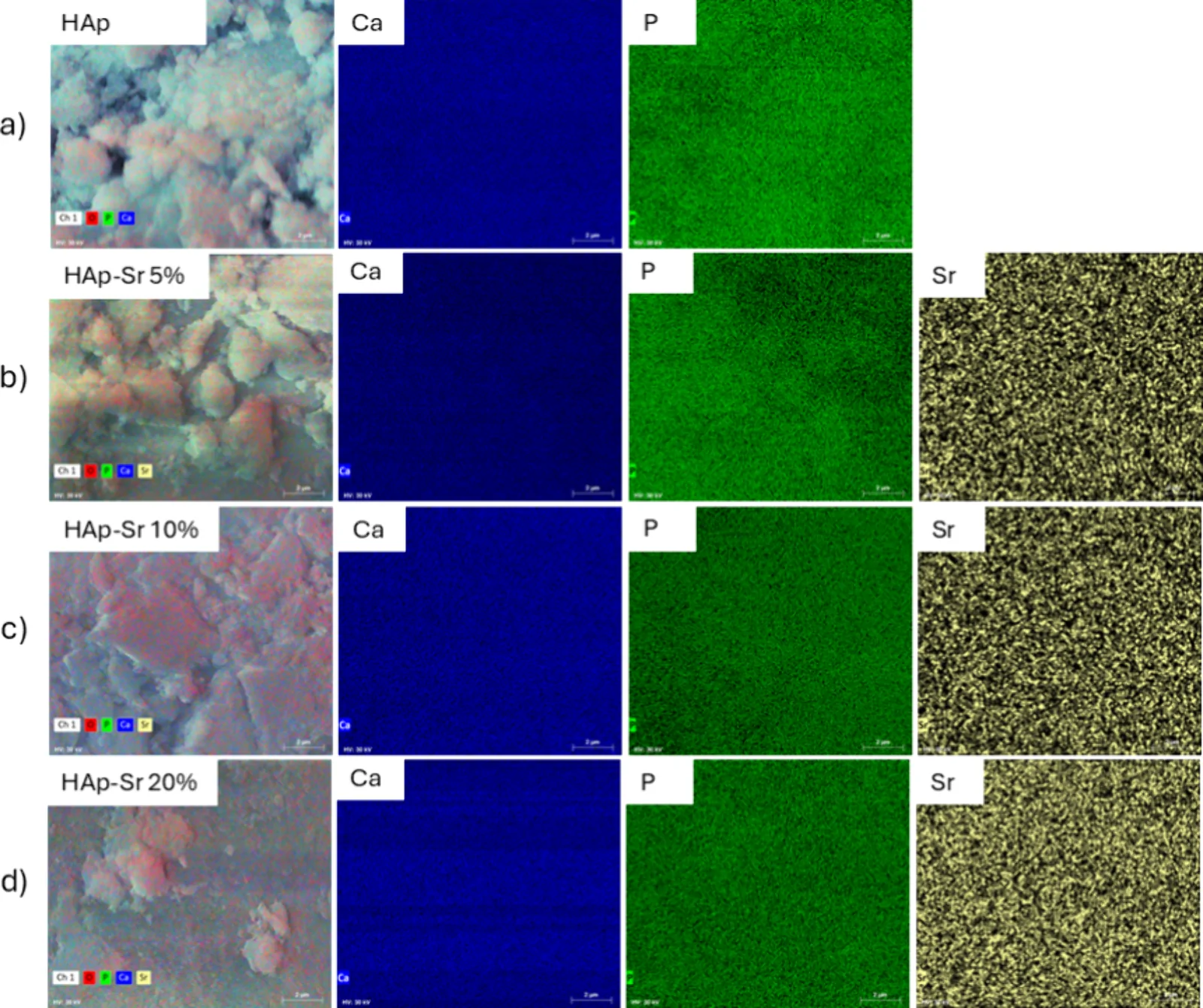
EDS elemental maps of HAp and Sr-doped HAp samples: (a) HAp, (b) HAp-Sr 5%, (c) HAp-Sr 10%, and (d) HAp-Sr 20%, showing the spatial distribution of Ca, P, and Sr. The Sr maps indicate a homogeneous elemental distribution without detectable segregation.

The semi-quantitative EDS analysis (Table 5) further supports these findings. The Sr molar content increased from 0.73% for HAp-Sr 5% to 1.81% and 4.45% for HAp-Sr 10% and HAp-Sr 20%, respectively. Accordingly, the Sr/Ca ratio also increased, reaching values of 3.6%, 8.2%, and 22.5% for HAp-Sr 5%, HAp-Sr 10%, and HAp-Sr 20%, respectively. These results demonstrate a clear correlation between the nominal and measured compositions, confirming the effectiveness of the doping process.

**Table 5:** Elemental Composition (mol %) of Sr-Doped Hydroxyapatite Samples Determined by EDS

**Element**	**HAp-Sr 5%** (mol %)	**HAp-Sr 10%** (mol %)	**HAp-Sr 20%** (mol %)
O	68.04(1.92)	62.10(1.94)	62.57(1.87)
P	10.97(46)	14.05(48)	13.23(43)
Ca	20.25(68)	22.03(76)	19.75(63)
Sr	0.73(10)	1.81(20)	4.45(44)
Sr/Ca (%)	3.6	8.2	22.5

Additionally, the Ca and P contents remained within expected ranges for hydroxyapatite, although slight variations were observed, which may be associated with local compositional heterogeneities inherent to the technique. This behavior also corroborates the values that approximate the intended content of Sr in all samples, given the technique limitations, and align with occupancies found in Rietveld refinement.

The results of elemental analysis (C, H, and N) for pure HAp and Sr-doped samples are presented in Table 6. The obtained values show that all HAp-based samples exhibit very low contents of carbon, hydrogen, and nitrogen, consistent with the essentially inorganic nature of hydroxyapatite. The slightly higher carbon content observed for pure HAp (0.7%), compared to the lower values found for the Sr-doped samples, suggests the presence of carbonate species in the undoped material. This observation is in agreement with the FTIR results, which indicated the presence of carbonate groups in pure HAp, while such features were significantly reduced or absent in the doped samples.

**Table 6:** CHN Analysis (wt %) of HAp and Sr-Doped Hydroxyapatite Samples^a^

**Sample**	C (wt %)	H (wt %)	N (wt %)
**HAp**	0.7	0.4	0.0
**HAp-Sr 1%**	0.1	0.2	0.0
**HAp-Sr 5%**	0.2	0.3	0.0
**HAp-Sr 10%**	0.1	0.2	0.0
**HAp-Sr 20%**	0.3	0.2	0.0

a All measurements were obtained with an estimated instrumental uncertainty of ±0.3%.

The decrease in carbon content with increasing Sr concentration further supports the interpretation that Sr incorporation into the HAp lattice hinders carbonate substitution, likely due to structural distortions and changes in the local chemical environment.

### 3.1 Stability Assay

The stability of Sr in the HAp matrix was evaluated by monitoring the release of Sr^2+^ ions from HAp-Sr samples (5%, 10%, and 20%) in simulated body fluid (SBF) after 24 and 48 h of incubation. The results are summarized in Table 7.

**Table 7:** Strontium Release from HAp-Sr Samples in Simulated Body Fluid (SBF)

	**Sr Release (mg L** ^**‑1**^ **)**		**Sr Release (%)**	
Sample	24 h	48 h	24 h	48 h
**HAp-Sr 5%**	3.37	3.37	7.91	7.91
**HAp-Sr 10%**	7.27	7.70	8.73	9.25
**HAp-Sr 20%**	2.94	2.94	1.84	1.84

Overall, all samples exhibited low Sr release, indicating a high stability of the dopant within the hydroxyapatite matrix. The HAp-Sr 5% and HAp-Sr 10% samples showed measurable ion release, with values of 3.37 mg L^–1^ and 7.27–7.70 mg L^–1^, respectively. In contrast, the HAp-Sr 20% sample exhibited lower release (2.94 mg L^–1^), deviating from the expected trend of increasing release with higher dopant content.

This behavior may be associated with structural effects induced by high Sr incorporation. Additionally, the lower release observed for HAp-Sr 20% may be related to the preferential release of Ca^2+^ ions, which tend to equilibrate with the Ca^2+^ already present in the SBF solution.^56^ Mocanu *et al*. reported increased release in SBF over 90 days for co-doped hydroxyapatite containing 5 and 10 at% Sr, which is consistent with the trends observed in the present study.^56^

Another possible explanation is the reprecipitation of Sr-containing calcium phosphate phases on the material surface during immersion in SBF. The presence of carbonate and phosphate ions in the medium can promote nucleation and growth of apatite-like phases, leading to reincorporation of released Sr^2+^ and, consequently, a reduction in its concentration in solution.^57^

The similarity between the values obtained at 24 and 48 h suggests that Sr release reaches a plateau within the first 24 h, resulting in a stable release profile over the evaluated interval, with concentrations falling within the reported therapeutic window (2–45 ppm).^56^

### 3.2 Mechanical assays

The mechanical properties of the samples were evaluated using pellets prepared by uniaxial pressing without a sintering step. This approach was adopted to assess the intrinsic behavior of the compacted materials under the applied processing conditions, while also minimizing energy consumption associated with high-temperature sintering. It is important to note that the absence of sintering may result in lower absolute mechanical values due to reduced densification;^58^ however, this methodology allows for a reliable comparative analysis between compositions.

Vickers microhardness testing and nanoindentation via atomic force microscopy were employed to evaluate the potential of the synthesized nanocomposites for this purpose. Hardness, fracture toughness, and elastic modulus values are in Figure 8a–c and Table 8.

**Table 8:** Mechanical Properties of HAp and Sr-Doped Hydroxyapatite Samples Obtained from Vickers Microhardness and Nanoindentation Tests

**Sample**	**Vickers Hardness (MPa)**	**Fracture Toughness (MPa·m**1/2**)**	**Elastic Modulus (GPa)**
**HAp**	357 ± 20	0.269	2.45 ± 0.45
**HAp-Sr 5%**	401 ± 20	1.288	6.69 ± 0.89
**HAp-Sr 10%**	415 ± 32	1.304	6.49 ± 0.72
**HAp-Sr 20%**	337 ± 26	0.410	3.13 ± 0.77

**Figure 8:**
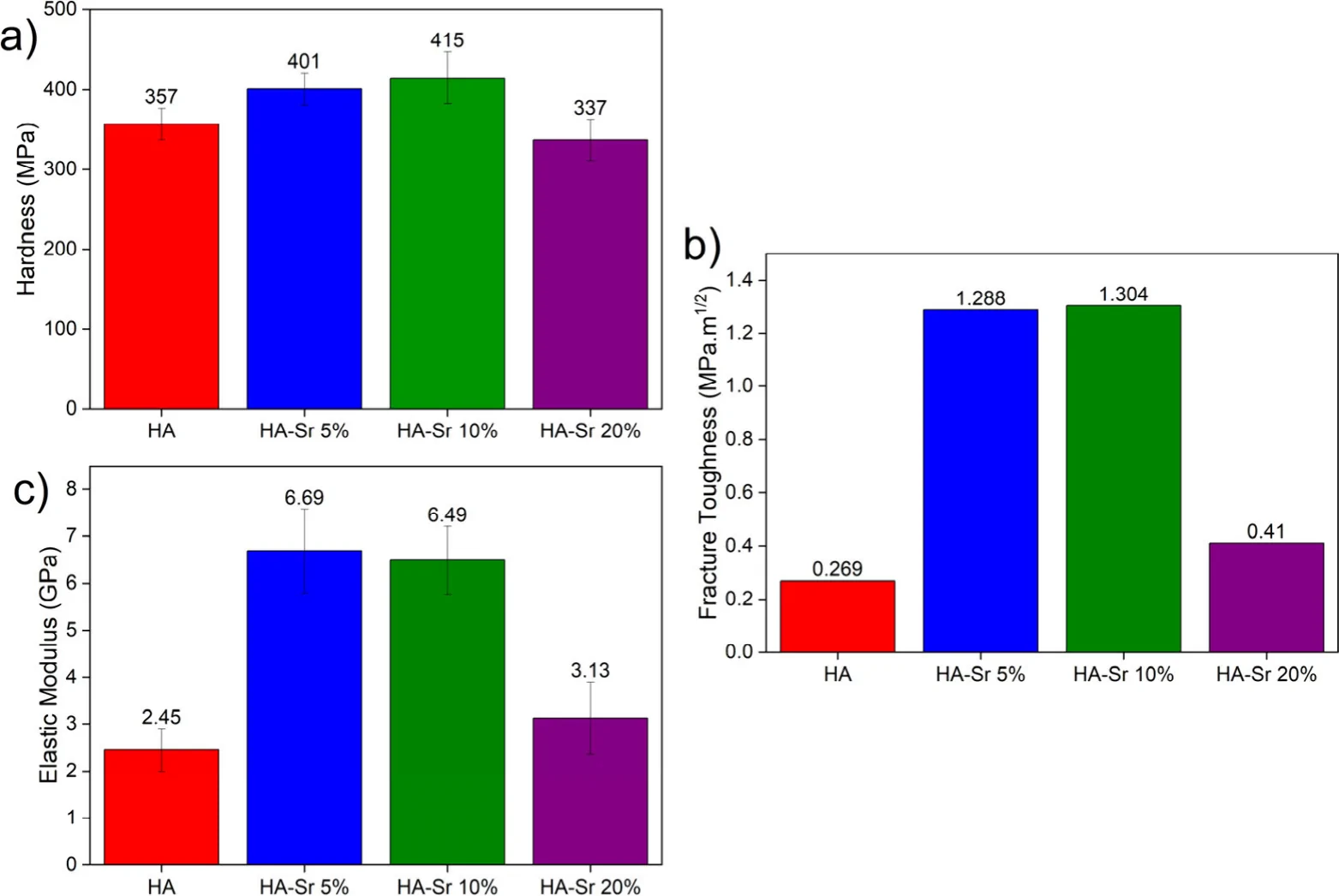
a) Vickers hardness, b) Fracture toughness, and c) Elastic modulus acquired via AFM nanoindentation.

The results indicated that incorporating strontium into the HAp matrix led to a noticeable improvement in mechanical properties, with Vickers hardness increasing by 12% for the 5% Sr sample and 16% for the 10% Sr sample. Literature reports that the hardness of human cortical and trabecular bone lamellae typically ranges from 234 to 760 MPa.^59^ In this context, the synthesized materials exhibited hardness values between 357 MPa for pure HAp and 415 MPa for the HAp-Sr 10% composition, placing them within the upper range observed for natural bone. Similarly, Klinkla *et al*. described an approximate 20% increase in hardness for HAp doped with Ag, Zn, and Mg, reaching values comparable to those obtained in the present work.^60^ The observed mechanical enhancement can be attributed to structural changes promoted by Sr incorporation, favoring a more efficient stress distribution and load transfer throughout the material.

Fracture resistance, estimated from indentation analysis supported by SEM observations, showed a substantial increase with Sr addition. Specifically, an improvement of 379% was obtained for the sample containing 5% Sr, while the 10% Sr composition exhibited a 385% increase compared to pure HAp. The resulting fracture toughness values approach those reported for cortical bone (∼2 MPa·m^1/2^), emphasizing the positive effect of Sr on the mechanical performance of the composites. This enhancement suggests greater resistance to crack initiation and propagation, likely due to more effective energy dissipation mechanisms introduced by Sr doping. Comparable results have been reported in the literature. Veljović *et al*. reported that strontium doping via two-step sintering resulted in increased fracture toughness (1.54 MPa·m^1/2^) along with high hardness, indicating improved mechanical performance for bone regeneration applications.^61^ Notably, the fracture toughness values obtained in the present work are of similar magnitude, despite the fact that the samples were not subjected to sintering. This represents an important advantage, as it demonstrates that significant mechanical enhancement can be achieved without the need for high-temperature processing, potentially preserving nanoscale features and reducing processing costs.

The elastic modulus values, extracted from the load–depth curves obtained by nanoindentation (Figure 9), show a significant increase from 173% and 165% for HAp-Sr 5% and HAp-Sr 10%, respectively. The increase can be attributed to lattice distortion and improved packing induced by Sr^2+^ substitution, which enhances the resistance to elastic deformation. Hassan *et al*. synthesized and obtained scaffolds where increasing the amount of Sr up to 4 wt % increased the elastic modulus by ∼290%, showing that the doping process is able to increase the resistance to elastic deformation of the material.^62^ It is valid to observe that a process to produce a scaffold with such an increase involves long step processes in relation to an improvement of 173%, obtained by only pressing the green powder.

**Figure 9:**
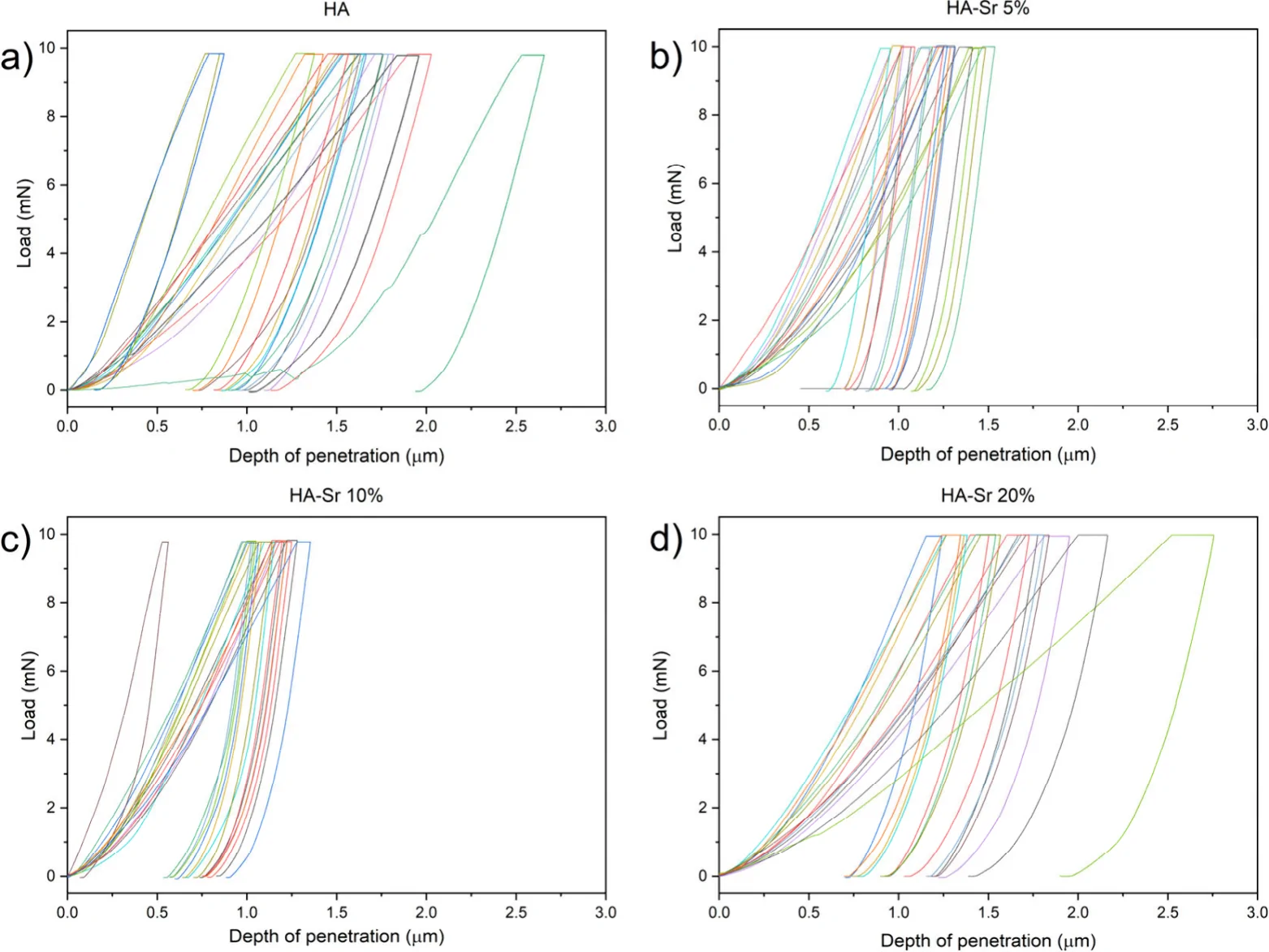
Load–depth curves obtained by nanoindentation for (a) HAp, (b) HAp–Sr 5%, (c) HAp–Sr 10%, and (d) HAp–Sr 20%. Multiple curves are shown for each sample, corresponding to different indentations.

The reduced enhancement observed for the HAp–Sr 20% sample is consistent with the overall mechanical behavior of the system, as also reflected in the microhardness and fracture toughness results. While moderate Sr incorporation (5–10%) promotes improvements in mechanical properties, the highest Sr content leads to a deterioration in performance, indicating that excessive substitution negatively affects the structural integrity of the material.^29^

This behavior can be associated with increased lattice distortion, which disrupts the crystal coherence, as evidenced by the pronounced unit cell expansion in the Rietveld refinement results and particle size reduction.^63^ In parallel, vibrational spectroscopy (FTIR-ATR and DRIFTS) reveals a significant reduction in the relative area of OH^‑^ bands and changes in phosphate vibrational modes, indicating the formation of local disorder or OH^‑^ loss, as seen in literature.^7^

Consequently, these structural changes reduce the material’s resistance to deformation and crack propagation, explaining the lower elastic modulus gain and the decline in hardness and toughness at 20% Sr. These results indicate the existence of an optimal Sr substitution range, in which the balance between lattice expansion and structural stability enhances the mechanical properties of the material, while higher concentrations lead to excessive distortion and reduced performance, consistent with the structural variations observed in the XRD, FTIR-ATR, and DRIFTS analyses.

From a biomedical perspective, the observed improvements in mechanical properties at moderate Sr concentrations are particularly relevant for bone tissue engineering applications.^64^ Materials with enhanced hardness and fracture resistance are desirable for supporting mechanical loads and resisting crack propagation in physiological environments. At the same time, the relatively lower stiffness compared to fully dense ceramics may be advantageous in reducing stress shielding effects, promoting better mechanical compatibility with natural bone.^65,66^

Overall, the results indicate the existence of an optimal Sr content for mechanical enhancement, with HAp-Sr 5% and HAp-Sr 10% - statistically similar - presenting the most balanced combination of hardness, fracture resistance, and stiffness. Among these, HAp-Sr 5% stands out as a particularly suitable composition, as it combines improved mechanical properties with reduced structural perturbation, as previously evidenced by FTIR and Raman analyses, and a more controlled Sr release profile. These characteristics make it a promising candidate for applications in bone regeneration and load-sharing biomaterials.

### 3.3 Ciprofloxacin Incorporation and Release Behavior

The HAp–Sr 5% composition was selected for ciprofloxacin incorporation based on its overall balanced performance among the investigated samples. This composition exhibited improved mechanical properties compared to pure HAp, while maintaining structural integrity and avoiding the pronounced lattice distortions observed at higher Sr contents. Spectroscopic and morphological analyses further indicated minimal structural perturbation and preservation of the characteristic nanorod morphology. In addition, its moderate and stable Sr release profile suggests suitable chemical stability for biomedical applications. These combined features make the HAp–Sr 5% system an appropriate candidate for evaluating drug incorporation and release behavior. For comparison purposes, pure HAp was also evaluated under the same conditions to establish a reference for the influence of Sr incorporation on drug loading and release behavior.

Elemental carbon analysis was used to estimate the amount of ciprofloxacin (CP) incorporated into the samples, and the results are presented in Table 9. Since negligible carbon content was detected in the non-loaded samples, the measured carbon fraction can be directly correlated with the amount of drug incorporated into the matrix.

**Table 9:** Ciprofloxacin Content and Mass Loss of HAp-Based Samples Determined by CHN and TGA Analyses^a^

**Sample**	**C (wt %)**	**CP Content (wt %)**	**Mass Loss (125–900 °C) (wt %)**	**Mass fraction (wt %)**
**HAp + CP**	29.7	49	46	45
HAp-Sr 5% + CP	22.0	36	38	38
**CP (pure)**	60.8	100	—	—

a CP content was estimated from CHN analysis. Mass loss corresponds to TGA results between 125 and 900 °C. Mass fraction corresponds to UV-Vis readings for the permeate that was not incorporated, obtaining the amount of CP incorporated in the samples

The HAp sample exhibited the highest drug loading (49 wt %), followed by HAp-Sr 5% (36 wt %). These results indicate that Sr incorporation reduces the capacity of the material to incorporate CP. This behavior can be associated with structural modifications induced by doping, particularly the reduction of OH^–^ groups, as previously evidenced by FTIR analysis. These groups are known to act as preferential interaction sites for drug adsorption, and their decrease limits the interaction between CP and the HAp surface.

Thermogravimetric analysis (TGA) was used to further confirm drug incorporation, as shown in Figure 10. The mass loss observed between 125 and 900 °C corresponds to the thermal degradation of ciprofloxacin and is consistent with the values obtained from elemental analysis, reinforcing the reliability of the incorporation results.

**Figure 10:**
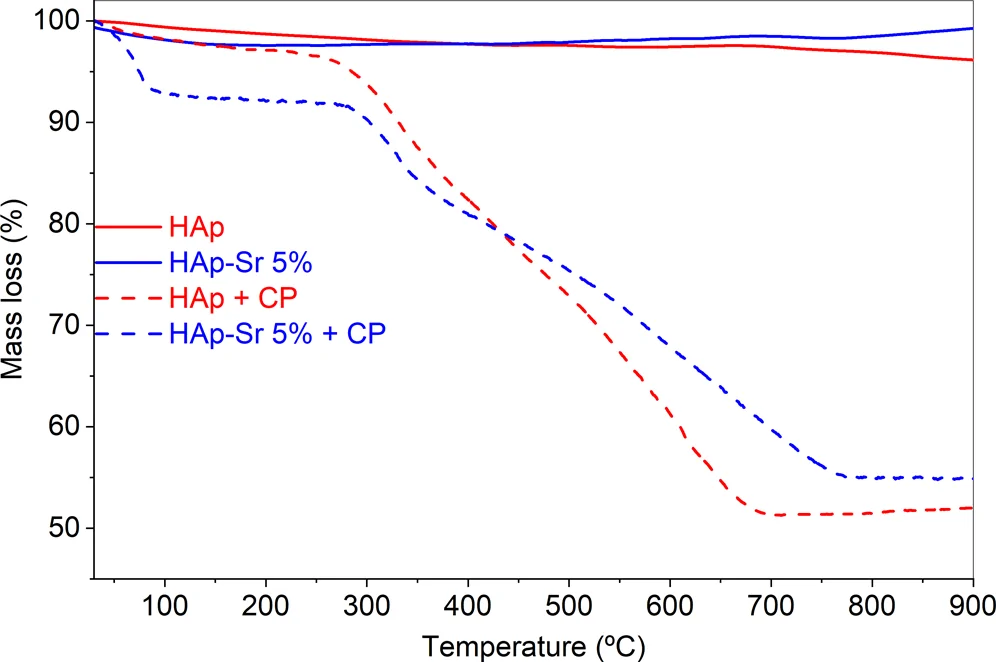
TGA curves of HAp and HAp-Sr 5% samples before and after ciprofloxacin incorporation, showing mass loss associated with drug degradation.

Considering the initial 1:1 mass ratio used during loading, the incorporation efficiency was calculated. The HAp sample showed the highest incorporation efficiency (92%), while the Sr-doped sample exhibited a lower value (∼76%), confirming that doping reduces drug uptake. Marinho *et al*. obtained a similar reduction in ciprofloxacin incorporation when using Eu-doped HAp and attributed that to OH^‑^ loss in its structure.^67^

The release profiles of CP in SBF are presented in Figure 11. All samples exhibited biphasic release behavior, characterized by an initial burst release followed by a slower and more sustained release stage. The initial stage can be attributed to the desorption of weakly bound drug molecules from the surface, while the second stage is associated with diffusion-controlled release from the interior of the matrix.^68,69^

**Figure 11:**
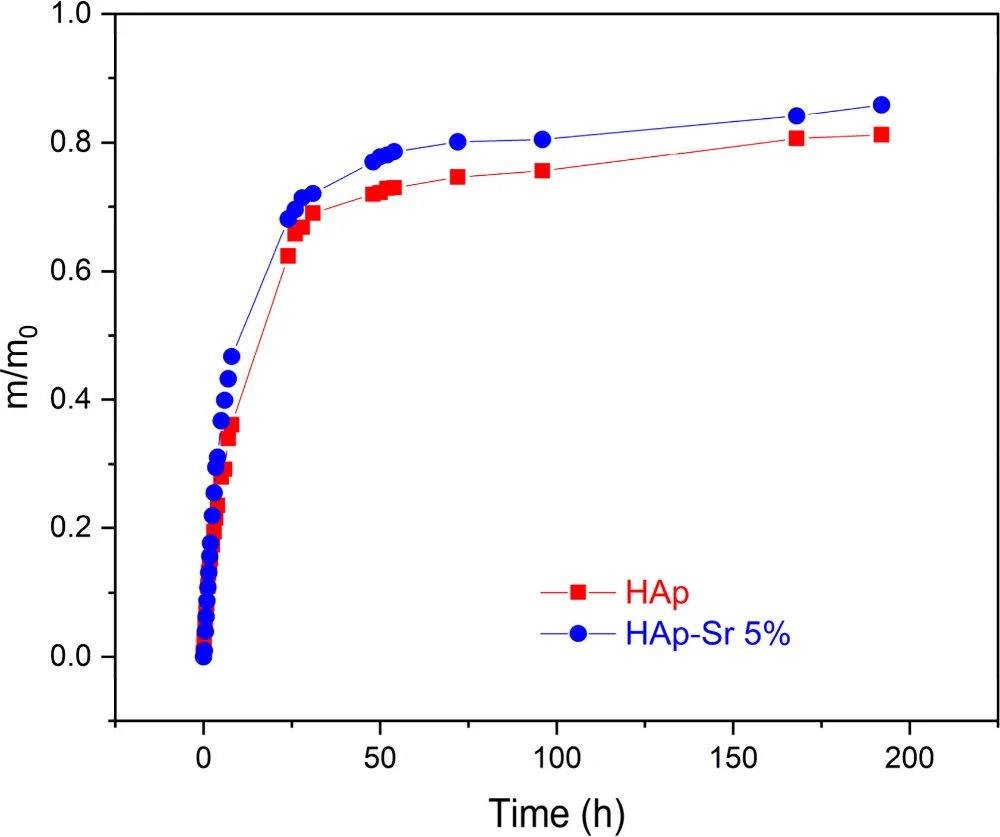
Drug release profile up to 192 h.

To better understand the release mechanism, the experimental data were fitted to different kinetic models, including zero-order, first-order, Higuchi, and Korsmeyer–Peppas models. The fitting graphs are shown in Figure 12, and the fitting parameters are summarized in Table 10.

**Figure 12:**
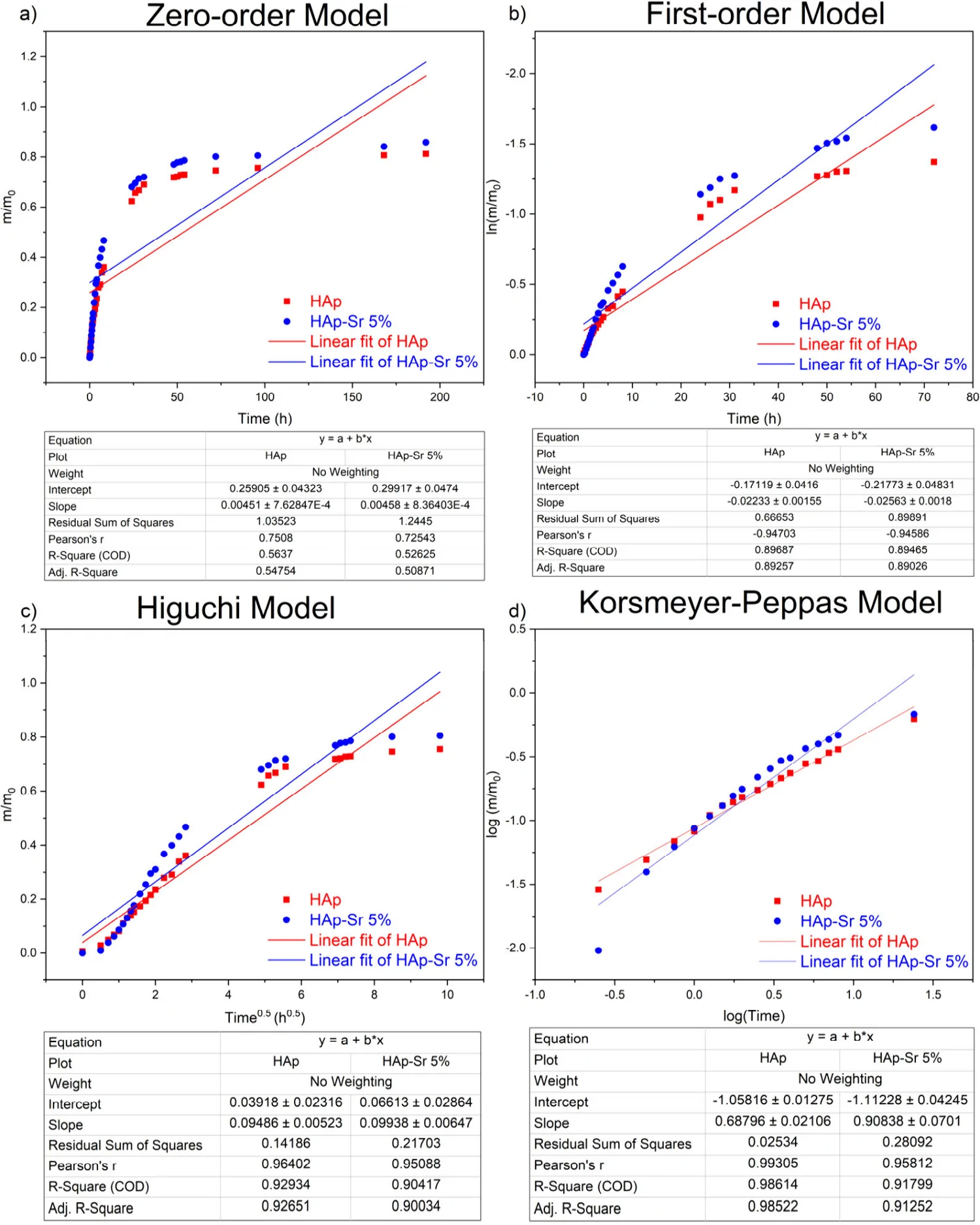
Drug release linear fitting for HAp and HAp-Sr 5% samples for a) zero-order model, b) first-order model, c) Higuchi model, and d) Korsmeyer-Peppas model.

**Table 10:** Kinetic Parameters of Ciprofloxacin Release for HAp-Based Samples

**Zero-Order Model** (M_t_/M∞ = K_0_t)			
**Sample**	**Equation**	**Adj**. **R** ^**2**^	Pearson’s r
HAp + CP	$M_{t}/M_{\infty }=0.004t$	0.55	0.751
HAp-Sr 5% + CP	$M_{t}/M_{\infty }=0.004t$	0.51	0.725

**First-Order Model** (M_t_/M∞ = 1 – e^‑K^1^t^)			
**Sample**	**Equation**	**Adj**. **R** ^**2**^	**Pearson’s r**
HAp + CP	$M_{t}/M_{\infty }=1-e^{-0.022t}$	0.89	–0.947
HAp-Sr 5% + CP	$M_{t}/M_{\infty }=1-e^{-0.026t}$	0.89	–0.946

**Higuchi Model** (M_t_/M∞ = K_H_t^1/2^)			
**Sample**	**Equation**	**Adj**. **R** ^**2**^	**Pearson’s r**
HAp + CP	$M_{t}/M_{\infty }=0.095t^{1/2}$	0.93	0.964
HAp-Sr 5% + CP	$M_{t}/M_{\infty }=0.099t^{1/2}$	0.9	0.951

**Korsmeyer–Peppas Model** (M_t_/M∞ = Kt_n_)			
**Sample**	**Equation**	**Adj**. **R** ^**2**^	**Pearson’s r**
HAp + CP	$M_{t}/M_{\infty }=11.43t^{0.68}$	0.99	0.993
HAp-Sr 5% + CP	$M_{t}/M_{\infty }=12.95t^{0.90}$	0.91	0.958

For HAp and HAp-Sr 5%, the Korsmeyer–Peppas model provided the best fit, with *n* values of 0.68 and 0.90, respectively, indicating a non-Fickian transport mechanism governed by both diffusion and structural relaxation processes.

Furthermore, the release concentrations fall within the range of minimum inhibitory concentrations (MIC) reported for ciprofloxacin (0.25–2 μg mL^–1^),^70^ indicating that the developed system is capable of delivering therapeutically relevant doses while minimizing excessive drug exposure. In a similar study, Venkatasubbu *et al*. observed that either pure or doped (Zn) HAp can be used as a drug delivery system for CP and attribute a faster release for doped samples due to the ion-dopant release in solution, in agreement with the results obtained in this study.^31^ Thus, the material demonstrates potential as a suitable candidate for the intended application, exhibiting the required characteristics.

Overall, the results demonstrate that the incorporation of ciprofloxacin endows the material with multifunctionality, combining structural support with controlled drug delivery, which is highly desirable for applications in bone regeneration and infection control.

## 4 Conclusions

Sr-doped hydroxyapatite nanorods were successfully synthesized via a hydrothermal route, and the effects of increasing Sr substitution on the structural, morphological, mechanical, and drug delivery properties were investigated. XRD analysis and Rietveld refinement confirmed the successful synthesis of single-phase hydroxyapatite and the substitutional incorporation of Sr^2+^ into the crystal lattice, without the formation of secondary phases. Increasing the strontium concentration promoted an expansion of lattice parameters and unit cell volume, accompanied by a shift of diffraction peaks toward lower 2θ values. Furthermore, progressive broadening of diffraction peaks was observed, indicating increased crystal lattice distortion and reduced particle size, while preserving the characteristic hexagonal crystal structure of hydroxyapatite. Sr content directly influenced mechanical performance. Moderate doping levels (5–10%) promoted improved hardness (up to 16%), fracture toughness (up to 385%), and elastic modulus (up to 173%), whereas higher Sr content (20%) led to performance degradation, indicating a clear doping threshold for maximizing the mechanical properties of hydroxyapatite. The results indicate that the Sr substitution level strongly influences the balance between structural integrity and mechanical performance, with intermediate substitution levels providing the most favorable overall behavior. The use of non-sintered pellets enabled a direct assessment of the intrinsic behavior of the compacted materials, providing a low-energy processing route while preserving comparative reliability across compositions. Stability assays demonstrated low Sr release in simulated body fluid, confirming strong dopant retention and suggesting suitability for applications requiring controlled ion delivery. Ciprofloxacin studies showed a biphasic release profile, with an initial burst followed by sustained release. Kinetic modeling indicated that drug release is governed by combined diffusion and structural relaxation mechanisms. Overall, the HAp-Sr 5% composition exhibited the most balanced performance, combining improved mechanical properties, structural stability, and controlled ion/drug release. These results show that controlled Sr substitution can be used to modify the structural, mechanical, and drug delivery behavior of hydroxyapatite, supporting its potential for load-sharing biomaterials and localized drug delivery applications.
